# The Management of Labour, Etc.

**Published:** 1886-05

**Authors:** 


### Book Reviews.

The Management of Labour and of the Lying-In Period. A Guide for the Young Practitioner. *By* Henry G. Landis, A. M., M. D., *Professor of Obstetrics and Diseases of Women in Starling Medical College, etc. 12 mo., pp. viii., 334. Philadelphia:* Lea Brothers & Co., 1885. *Chicago:* Jansen, McClurg & Co.

The aim of this little volume is to serve as a guide to practice, divested of all superfluous or irrelevant details. The author desires " modestly to say to the physician ":

" Let this not lie unread upon the shelf, unless you know some better way yourself." The design of the book is certainly good. But the American press is prolific of such books,—some of them very excellent treatises. The practitioner justly demands of the author by what authority he doeth these things. Unless the author can meet this challenge, he is presumptuous to offer a new book to the profession. Let us accordingly inquire as to the "manner, skill, and ability with which the design has been carried out."

In the chapter on "The Normal Standard," the writer asserts that in ideally normal labour there is no pain, or but little, and that six hours represent the maximum limit of normal parturition. In the discussion of the diagnosis of presentation no allusion is made to abdominal palpation. Dr. Landis says the great objection to frequent vaginal examinations is that the finger is apt to withdraw each time some of the lubricating mucus from the vagina. The freedom with which the author advises digital dilatation of the *cervix uteri*, and his suggestions with reference to the care of the perineum, evinces an utter disregard of principles of treatment definitely established early in this century. The cord must be tied as soon as the child cries! The author usually "Credê's the placenta!" "There is no reason why we should not use the verb *to Credê* as simpler than the periphrasis 'to use the method of Credê.'" It is recommended that within a few hours after the termination of labour, the woman "sit upon a chamber placed in the bed and try to urinate." "In puerperal eclampsia," says Dr. Landis, "the temperature increases with each convulsion, and in fatal cases may reach 100° F. or 109° F. In uræmia the temperature falls and may be subnormal."

In the chapter on pelvimetry, we are informed that "external pelvimetry is of no use in an individual case." It is needless to multiply examples of grave errors both as to fact and opinion. There is scarcely a single page which does not absolutely condemn the treatise. The mode in which the design of the work has been carried out is hopelessly bad.

An eminent obstetrician recently said to an acquaintance, "The practice of midwifery in the United States is rapidly becoming one of the lost arts." If the little volume before us be regarded as a fair exponent of the practice of obstetrics in the United States, we would be compelled to adopt the opinion of our pessimistic friend. We are under the impression, however, that the author's views and methods of practice are peculiarly his own.

